# Supplementary material for: VAB-8/KIF26, LIN-17/Frizzled, and EFN-4/Ephrin control distinct stages of posterior neuroblast migration downstream of the MAB-5/Hox transcription factor in Caenorhabditis elegans
Source: PLoS Genet. 2025 Oct 10;21(10):e1011906. doi: 10.1371/journal.pgen.1011906 (PMC12533845; doi:10.1371/journal.pgen.1011906)
Supplement: Attachment — Submitted filename: PLoS Genetics review 2025.docx [file pgen.1011906.s005.docx]

**PLoS Genetics-D-25-00704 review**

**07.15.2025**

This manuscript follows up on a paper published by the Lundquist lab in 2024, which used RNA sequencing of Q-lineage cells in mab-5 gain-and loss-of-function mutants to identify genes under mab-5 transcriptional regulation, in the hope of identifying QL neuroblast lineage-specific genes. In the current manuscript, the authors home in on the atypical kinesin vab-8, frizzled protein lin-17, and ephrin efn-4 as candidate transcriptional targets of mab-5 (originally identified in their 2024 paper). Using genetic analysis, the authors reveal that all three targets function genetically downstream of mab-5. In addition, the authors categorize their contribution to QL descendent cell migration into three distinct phases, with vab-8 having roles in all three phases, lin-17 in phases two and three, while efn-4 is only required for the final cell migration of QL.ap (which will become the PQR neuron) to its terminal location. In support of these data, the authors also performed time-lapse imaging of cell migration defects in vab-8, lin-17, and efn-4 mutants (and others). This revealed some interesting details of QL.ap neural development. If cell migration of QL.ap failed, the cell entered terminal differentiation 1-2 hours early (as defined by the premature extension of a dendrite growth cone), suggesting active suppression of terminal differentiation when the cell body is migrating. Additional cell migration assays and timelapse data on supporting mutants (such as mab-20) nicely round out the study. The authors also publish new mutant alleles that were identified in forward genetics screens to identify novel genes required for QL migration. Overall, this is a thorough and well-executed manuscript with a strong body of genetic analysis, featuring multiple alleles of each gene in question, in all appropriate combinations, and with generous n-numbers, and appropriate statistical analyses. The use of genomic sequencing to clarify the nature of the mab-5(e1751) tandem duplication (an apparent gain-of-function allele) was another nice touch. Note that the RNAseq data upon which this work is based suffers somewhat from overly stringent q-value interpretations/corrections for multiple comparisons, which renders some of the transcript level differences as not-significant when statistically tested as such. Despite this, the authors use of Q-lineage reporter genes in their various genetic backgrounds thoroughly validates the RNAseq data and removes any concern from their RNAseq conclusions.

No major revisions are necessary from this reviewer’s point of view. If the following minor comments are addressed, this manuscript will be of a quality and significance appropriate for publication in PLoS Genetics.

Minor comments/typos:

P6 (Abstract) typo, “Surprisingly, failure of stages two and three….”

P8, first paragraph, “Hox “realizator” genes…” Why not just state “Hox transcriptional targets”?

P9, penultimate paragraph, typo “which acts with efn-4 in prevention…” Also, “posterior QL lineage…)

P10 last paragraph, “expression of LIN-17/Fz…”

P12, paragraph 2. Consider changing this sentence to “AQR migration was unaffected in these mutants (Table 2).” to improve readability.

P13, subheading: state “vab-8, lin-17, and efn-4 lof mutants suppress mab-5 gof”. This will clarify the meaning of this subheading.

P17, penultimate paragraph, first sentence, “QL.a is divided”; delete “is”.

P20, penultimate paragraph, last sentence, “are required for to complete…” delete “for”.

P21, first full paragraph, first sentence, typo: “lin-7” should be “lin-17”. Second sentence, typo: “lin-17(e145)” should be “lin-17(e1456)”.

P21, last paragraph, typo: “ev754” should be “ev574”. Also, the Culotti et al. 2000 reference should be in parentheses.

P23, Materials and Methods, Genetics: The authors state that various transgenes generated in their lab were integrated into the genome. Please clarify how this was done, and whether the integrated transgenes were outcrossed prior to use.

P31, second paragraph: “QL,ap” should be “QL.ap”

P31 third paragraph, second sentence: “VAB-8 is a conserved…” Consider breaking this sentence into two for improved readability.

P31, last sentence. Add a period, not a comma.

P33, figure 1 legend. “lqIs244[Pgcy-32::gfp] -32::GFP” - delete underlined section. Also, please clarify if this is a CFP or GFP reporter gene, and be consistent throughout the text.

P33 figure 2 legend. **A]** should be A). lqIs220 (last sentence) should be italicized.

P35 figure 4 legend. “imagijg” should be “imaging”. Figure 5 legend, “hous” should be “hours”

P37 Acknowledgements: “component” should read “a component”.

P39, references. The Knop et al., Kratsios et al., Lu et al., Paolillo et al., So et al., references appear incomplete.

P42, table 1. The mab-20 q-value (last row of the table) is less than 0.05 but is reported as not significant. Typo?

P43, table 2, suppression experiments: the PQR migration data for mab-5(e1751) doesn’t add up. Typo?

P46, table 4. Would it be possible to clarify what “non-position 4” means (RH column)? Is it position 5, or is it anywhere, else other than position 4? Also, last row of table 4, “mom2…” should read “mom-2…”
