# Supplementary material for: VAB-8/KIF26, LIN-17/Frizzled, and EFN-4/Ephrin control distinct stages of posterior neuroblast migration downstream of the MAB-5/Hox transcription factor in Caenorhabditis elegans
Source: PLoS Genet. 2025 Oct 10;21(10):e1011906. doi: 10.1371/journal.pgen.1011906 (PMC12533845; doi:10.1371/journal.pgen.1011906)
Supplement: Attachment — Submitted filename: Response to Reviewers #2.docx [file pgen.1011906.s007.docx]

Reviewers 2 and 3 were satisfied that the work was suitable for publication.

We followed the academic editor’s suggestion and removed “*mab-5* target” throughout the manuscript as described below.

We address the comments of Reviewer 1 below:

Reviewer #1: The revised manuscript does not adequately address the major concerns raised by this reviewer and by reviewer #2. Aside from incorporating publicly available ChIP-Seq data, the authors have not performed additional experiments and have not been responsive to requests to temper or adjust their claims.

In my view, it is not justified to describe lin-17, vab-8, and efn-4 as “MAB-5 targets” based solely on relatively weak RNA-seq data and their genetic requirement for a mab-5 gain-of-function phenotype. The authors offer several possible explanations for their inability to detect changes with the reporter constructs suggested by reviewer #2, but the most straightforward explanation is that these genes are not directly regulated by MAB-5. This concern is particularly relevant for efn-4, for which no binding sites are present.

We no longer refer to these genes as “MAB-5 targets”. We will reference the fact that these genes show significant differential regulation in some *mab-5* mutant backgrounds, in the case of *vab-8* in all *mab-5* mutant backgrounds. We use the term “regulated by *mab-5*” in the manuscript instead. We note thar reviewer 2 was satisfied that the manuscript was suitable for publication.

Other important issues also remain unresolved, such as the interpretation of the double mutant phenotypes and the weak cell-autonomous rescue. I do not agree with the explanations the authors provide. For instance, their suggestion that the heterologous promoter may not drive expression at the correct time is inconsistent with their own imaging data, which clearly shows robust expression at the appropriate place and stage.

Our genetic results are that no strong synergy is found, suggesting that they do not act redundantly with one another. Our explanation on pp.17-18 is that this lack of genetic synergy suggests the molecules could act together rather than in parallel pathways.

The *egl-17* promoter is expressed throughout Q migration. Our argument on pp. 12-13 is that there might be a precise time and/or level of gene function needed, and that continuous expression might not be suitable for rescue. We also mentioned that the genes also might act non-autonomously in tissues outside of the Q cells. These are plausible scenarios that are often invoked to explain partial transgenic rescue, which is quite common using heterologous promoters.

Finally, the authors remain unresponsive even to minor, easily correctable points. Examples include their decision to retain the RNA-seq table despite reviewer #2’s request for its removal,

We argued why we think the table is important. In comments below, reviewer #2 did not object to our leaving the table in. We reiterate our arguments for leaving the table in: for the sake of transparency and so that readers can see the DE data first-hand without having to go digging through Paolillo et al., 2024.

and the continued claim in the abstract that “VAB-8 traffics distinct molecules to the plasma membrane,” despite my previous comment that VAB-8, as an immotile motor, cannot serve such a trafficking role.

We agree that VAB-8 is not a motile motor and said so in the manuscript (p. 25). We also referenced work that shows that VAB-8 is required for localization of molecules to subcellular regions despite not being a motor itself. We now avoid using the word “trafficking” in the manuscript, as this term risks being equated with motor activity. Instead we use “localization to the plasma membrane” in describing our model, which is consistent with previous referenced work.

Reviewer #2: I have reviewed the revised manuscript and find that my concerns have been addressed.

Reviewer #3: The authors have appropriately addressed and corrected essentially all of the minor revisions flagged in the first version. This is now a well written and significant body of work, suitable for publication in PLoS Genetics.
