# Supplementary material for: VAB-8/KIF26, LIN-17/Frizzled, and EFN-4/Ephrin control distinct stages of posterior neuroblast migration downstream of the MAB-5/Hox transcription factor in Caenorhabditis elegans
Source: PLoS Genet. 2025 Oct 10;21(10):e1011906. doi: 10.1371/journal.pgen.1011906 (PMC12533845; doi:10.1371/journal.pgen.1011906)
Supplement: Attachment — Submitted filename: Response to reviewer comments.docx [file pgen.1011906.s006.docx]

**We respond to each of the reviewer comments below in blue. We have revised the manuscript as the reviewers suggest.**

**We added an analysis of the modENCODE ChIP seq data for MAB-5 as requested. *vab-8* and *lin-17* have nearby MAB-5 ChIP seq peaks, but *efn-4* does not. These ChIP seq data were from embryos, L2, and L3 larvae, whereas Q cells migrate in L1. Furthermore, these ChIP seq data are from whole-animal preparations. Both of these factors might explain why *efn-4* did not show significant MAB-5 peaks in these data.**

**Reviewers' comments:**

Reviewer's Responses to Questions

**Comments to the Authors:
Please note here if the review is uploaded as an attachment.**

Reviewer #1: This manuscript investigates how the Hox gene mab-5 regulates posterior neuroblast migration in C. elegans via three candidate downstream effectors: vab-8/KIF26, lin-17/Frizzled, and efn-4/Ephrin. Using RNA-seq of Q-lineage cells, live time-lapse imaging, genetic mutants, and rescue constructs, the authors propose that these genes function at distinct, sequential stages of QL.ap migration. The main strength of the study is the methodological phenotypic characterization of the three mutants, as well as the rigorous assessment of several alleles for each gene. However, several gaps in the analysis remain, which would require significant revision before publication.

Major Points:
1] The data do not strongly support that the three genes are mab-5 targets. The changes reported in RNA-seq are not very strong or consistent across the lof and gof alleles. In general, RNA-seq data need to be validated by methods such as transcriptional reporters or qRT-PCR.

The inconsistent changes in expression in the lof and gof alleles was likely due to limitations of the bulk Q cell sorting and sequencing technique, as discussed in Paolillo et al, 2024. Variability in timing of expression, levels of expression, and sequencing both QL and QR lineages could all limit the resolution of this approach. In addition, variabilities between the three biological replicates could also obscure expression changes. In particular, *efn-4* was only necessary for the final third stage of migration. It is possible that RNA from the QL.ap cell was underrepresented in the sorted Q lineages given the starvation method to isolate Q cells. It is also possible that the expression of *efn-4* is transient in nature, which cannot be caught through bulk RNA-seq of *lof* or *gof* alleles as done by Paolillo et al. 2024.

Given the fact that we have a functional genetic assay to test if these genes act downstream of *mab-5,* we chose to cast a wide net. In this work, *vab-8*, *lin-17*, and *efn-4* were each shown to act genetically downstream of *mab-5*. We have added a caveat in the manuscript:

Inconsistency of expression changes across genotypes could indicate that these are not *bona fide* MAB-5 target genes. However, variability in the FACS sorting and bulk RNA-seq approach could also explain inconsistencies. For example, some genes might be expressed at specific times or in specific cells, and slight variations in the timing of Q cell isolation in biological replicates could limit ability to identify these genes consistently.”

Related to the previous point: could the authors analyze (at least in silico) the promoter regions of lin-17, vab-8, and efn-4 to test whether they contain mab-5 binding sites?

We did this analysis. ChIP-seq data from the modEncode project displayed on jBrowse on Wormbase indicate that MAB-5 has strong interacting regions in the adjacent to *lin-17* and *vab-8*. Thus, MAB-5 likely binds to chromatin adjacent to *lin-17* and *vab-8* promoters. No predicted MAB-5 binding sites were adjacent to *efn-4.* These ChIP-seq data were derived from whole animal preps of embryos, L2, and L3 larvae. Q neuroblast migration occurs in early L1 embryos, not yet represented in these data. Furthermore, whole animal preparations were used. If the interaction of MAB-5 with *vab-8* and *efn-4* promoters is transient in L1 or specifically in Q descendants and not all tissues, these interactions might not be detected. This is now discussed in the manuscript, and a Supplemental Figure 1 is including showing the MAB-5 ChIP-seq peaks around the three genes.

“modENCODE MAB-5 CHiP-seq data on Wormbase from embryos, L2, and L3 larvae (Gerstein *et al.* 2010; Sternberg *et al.* 2024) were analyzed for potential MAB-5 binding sites. *vab-8* and *lin-17* displayed significant peaks near the 5’ ends of the genes and isoforms (Supplemental Figure 1). *efn-4* showed no significant peaks. These data suggest that MAB-5 might act at the promoters of *vab-8* and *lin-17* but not *efn-4*. However, Q migration occurs in the L1 larva, which was not represented in this data. Any transient or cell-specific interactions of MAB-5 with the *efn-4* region might not be apparent in these data.”

2] The conclusion about cell autonomy could be strengthened, given that the rescue was weak. How many lines were tested? Have the authors compared their rescue to one in which vab-8 and efn-4 are expressed under their own promoters or a promoter that is more broadly expressed?

Three independent rescue lines for *efn-4*, *vab-8,* and *mab-20* were scored as described in Materials and Methods, now indicated in Table 2. Partial rescue is a common result in many heterologous promoter transgenic rescue experiments. Partial rescue is discussed in the results and the discussion sections, specifically issues of timing and levels of expression using heterologous promoters. These results show that these genes can at least in part function autonomously in the Q cells. Combined with the expression in Q cells and genetically acting downstream of *mab-5* in the Q cells, these studies make a strong case that these genes act downstream of *mab-5* in the Q cells. Studies using different promoters to get at the nature of this partial rescue are beyond the scope of this work.

3] I was unable to understand the interpretation of some of the double mutants. Particularly in the case of lin-17 and vab-8, the effect seems additive and would support them functioning in separate pathways. In any case, the authors’ conclusion that “these genes act in the same pathway or in independent pathways” is not informative because it proposes two interpretations that are the opposite of each other.

The main point of this was to highlight that no evidence of genetic synergy was detected in any double mutant combination. Genetic synergy would indicate that the genes act in parallel, redundant, compensatory pathways. This is not what was observed. Our results are consistent with the genes either acting in the same pathway or acting independently (not in parallel). The following change was made to explain these results:

“*vab-8, lin-17,* and *efn-4* might define parallel, redundant pathways that regulate posterior migration. Genetic synergy, where the phenotype of double mutants is significantly stronger than the additive effects of the single mutants alone, is indicative of redundant, parallel pathways. However, double mutants of *vab-8, lin-17,* and *efn-4* showed no synergistic genetic interactions in PQR migration (Table 1). The number of PQRs at position 4 generally resembled the single mutants alone. While there were fewer cells at *wild-type* position 5 in double mutants, this could be an additive effect of cells that migrated anteriorly in *vab-8* and *lin-17* single mutants. The lack of synergistic genetic interactions suggest that these genes do not define parallel, redundant pathways and instead might act together.”

4] The authors propose that VAB-8/KIF26 functions to traffic another molecule (abstract and discussion). This conflicts with several papers from mammals and worms that show that VAB-8/KIF26 is immotile. They should suggest a different model, one that is consistent with the literature.

Indeed, VAB-8 does not display microtubule motor activity but does bind to MTs and localizes to MT plus ends at synapses (Balseiro-Gomez et al., 2022). VAB-8 also affects trafficking along synapses and might cause dynein pausing at synapses which might regulate cargo delivery (Balseiro-Gomez et al., 2022). Thus, despite not being a MT motor itself, it does regulate MT trafficking, which is what our model was based on. This has been more thoroughly discussed in the manuscript:

“VAB-8 can bind to microtubules but lacks microtubule motor activity (Balseiro-Gomez *et al.* 2022).”

“*In vivo*, VAB-8 localizes to microtubule + ends at synapses, affects vesicle trafficking along microtubules, and might regulate cargo delivery to synapses by pausing Dynein (Balseiro-Gomez *et al.* 2022). VAB-8 is also involved in gap junction formation and maintenance (Meng *et al.* 2016). Consistent with a potential role in trafficking, the VAB-8 long isoform is required for the cell surface localization of the SAX-3 receptor in the ALM neuron (Watari-Goshima *et al.* 2007).”

Minor Points:
The manuscript is long and hard to read. It would benefit from shortening and editing.

Many of the results are complicated and require proper explanation and discussion. Every attempt has been made to make the manuscript readable without sacrificing the complexity of the results and their ramifications.

In the abstract: “Surprisingly” – correct spelling.

Done.

Consider adding a diagram of the proteins and the mutations for readability.

We considered this. These are well-studied and annotated genes and molecules and we do not think that a diagram is necessary. It would further lengthen and clutter the manuscript.

It was unclear to me how the data support a role for lin-17 both upstream and downstream of mab-5. This is not a major point, but if the authors have reporters that could show them affecting each other’s levels, that would be helpful.

This argument is a functional one. *lin-17* acts in canonical Wnt signaling to activate *mab-5* expression. Thus, *lin-17* mutants have anterior PQR migration similar to *mab-5.* Here it is shown that heterologous *mab-5* expression using the *egl-17* promoter can rescue anterior PQR migration (can rescue canonical Wnt signaling upstream of *mab-5)* but cannot rescue failure of posterior PQR migration also observed in *lin-17* mutants (downstream of *mab-5*). Thus, *lin-17* functionally acts both upstream and downstream of *mab-5.*

Reviewer #2: In this manuscript, the authors characterize the role of three genes, vab-8/kif26, lin-17/Fz and efn-4/Ephrin, in migration of a Q neuroblast (QL) and its descendants in C. elegans. These genes were identified in a previous study that used RNAseq on FACS-sorted Q cells to identify differentially regulated genes in mab-5/Hox gain and loss of function mutants. The authors further contribute to our understanding of QL migration through time-lapse studies showing that QL descendants reach their final positions in the tail through three distinct stages, each characterized by a pause and lamellipodia formation. They demonstrate that vab-8, lin-17, and efn-4 mutants exhibit PQR migration defects, with the interesting finding that they act at distinct stages of QL descendant migration. The authors show that these genes act cell autonomously and conduct detailed genetic analyses using double mutants and mab-5 gain-of-function suppression experiments, with results consistent with their function as downstream targets of MAB-5.

Overall, this manuscript is mostly well written (see comments below) and presents some novel insight into cell migration using the well-established Q neuroblast migration model. In particular, they characterize, what I believe are the first downstream targets of MAB-5/HOX in QL cells, VAB-8, LIN-17, and EFN-4 and show that distinct transcriptional programs are activated at specific stages during the long migration of Q descendants from the mid-body to the tail.

I find the methodology to be sound and the results convincing. However, I feel that the manuscript could have gone a bit further to describe what one or more of these genes are doing to promote QL migration at one or more of the three stages. The Discussion section raises the possibility that VAB-8 may regulate the trafficking of LIN-17 and/or EFN-4 to the membrane, or that EFN-4 may play a role in promoting lamellipodial protrusions. It seems like an omission not to test some of these ideas in this study. For example, the manuscript would be greatly strengthened by including one or more of the studies outlined below. That said, it may also be reasonable to argue that these experiments are not essential to support the main conclusions or are not easily performed due to confounding factors.

1. While I find the data that these genes are expressed in QL cells strong and convincing, I was surprised that the RNA-seq data was not validated through the most direct means of determining if the expression of vab-8, lin-17, and efn-4 from either GFP (or some other fluorophore) transcriptional/translation reporters or endogenously tagged genes is downregulated or off in mab-5 mutants. For example, this could be performed with reporters such as wyEx450(Plin-17::lin-17::GFP) (Klassen and Shen, 2007), juIs109 (EFN-4::GFP) (Chin-Sang et al., 2002), and vab-8p::VAB-8::GFP (Wolf et al, 1998). It would also be interesting to see if these genes are expressed dynamically during QL migration, given the saltatory nature of the migration. For example, is LIN-17 present early to initiate QL posterior migration through the wnt canonical pathway, but then downregulated and later upregulated at the second and third stages of migration. In the discussion, it was suggested that mab-5 may be acting to maintain lin-17 expression. This might be testable by examining the expression from wyEx450(Plin-17::lin-17::GFP) or a similar reporter in a mab-5 mutant background.

Experiments such as these immediately came to mind, and they were pursued. It would be great to show expression in Q cells dependent upon *mab-*5 as well as potential dynamics of expression. However, expression in Q cells could not be reliably and consistently observed from transcriptional reporters for *efn-4, vab-8,* and *mab-20.* Expression was likely too faint to observe, and expression in the posterior gut from many of these transgenes obscured the Q cells. The *efn-4* transgene *juIs109* was obtained, but it also showed no obvious Q cell fluorescence. Much time and effort could be spent identifying the correct conditions, transgenes, or genome edits to conduct these studies. RNA-seq indicates that these genes are expressed in the Q lineage, and we will continue to search for the right conditions, but don’t want to slow publication of these results. Instead, this work focuses mainly on the functional interactions between these genes.

2. A polarized (oriented toward the posterior) lamellipodia forms at each stage of migration. The time lapse studies (using one of the available Q cell membrane markers) could be strengthened by also showing how vab-8, lin-17, and/or efn-4 mutants affect lamellipodial dynamics or polarity at each stage. For example, do lamellipodia polarize normally but fail to initiate migration in a timely manner? Are there differential effects? In the 3rd stage, the cell body undergoes somal translocation. Is this process differentially affected in the three mutants?

We show that in mutants, lamellipodia fail to form and instead premature dendritic protrusions form. Defects in polarity of protrusion of lamellipodial or dendrites were not observed. Questions about the cell biological nature of this decision and of the cytoskeletal mechanisms of protrusion formation are interesting but we feel beyond the scope of this work.

3. The model presented in Fig 7 suggests that VAB-8 may be involved in trafficking LIN-17 and EFN-4 to the plasma membrane to promote migration. This model would be greatly strengthened if there was evidence that LIN-17 and/or EFN-4 were mislocalized in vab-8 mutants. These studies may be possible with some of the reporters mentioned in comment #1.

Again, these experiments immediately leapt to mind and were pursued, but as-of-yet unsurmountable technical difficulties in imaging of expression in Q cells arose. Solving these would greatly delay publication.

There are also a few minor Issues:

1. The manuscript contained numerous typos and possible sentence structure errors, some of which I have listed below for clarity (it would be helpful to include page and line numbers).

The manuscript has been proof-read again to identify typos.

A. Abstract – why say First, if there is no Second of Third point…
B. Intro – paragraph 1 - remove word ‘is’
C. Paragraph 2, egl10/wnt – should this be egl-20.
D. ‘Paradigm’ is mentioned in Fig 2B but not defined in the results section until much later in the results. When reading figure legend, did not know what paradigm referred to.
E. mab-20/Semaphorin, which acts with efn-4 n prevention of male tail
F. Figure 1 legend: ….. different genotypes using the lqIs244[Pgcy-32::gfp] -32::gfp]

All of these have been corrected. Paradigm has been removed from the Figure 2 legend.

2. I’m not sure if double mutant is the correct term to describe a single mutant in a transgenic background, for example: In vab-8(ev411); Pegl-17::mab-5 and vab-8(e1017); Pegl-17::mab-5 double mutants….

Agreed. “Double mutants” has been removed and replaced with the genotypes.

3. The first section of the results dealing with Table 1 is probably not necessary as these are findings from the previously published study and were already mentioned and cited in the introduction.

We prefer to keep these data in the manuscript as they provide a full and transparent record of the RNA-seq results for these genes, including the variability.

4. I believe one of the studies by Wightman et al. 1996 or Wolf et al. 1998 showed that vab-8 mutants had PQR migration defects. This should probably be mentioned again in the results section when the vab-8 PQR migration defect is described.

Indeed, Wightman et al, 1996 reported PQR migration defects in *vab-8* mutants, with PQR not located in the final position behind the anus. We reference this in the text:

“*vab-8* was previously reported to affect PQR migration (Wightman *et al.* 1996).”

5. In Table 3, I wasn’t quite sure at first what was meant by ‘1 and WT’. It took me a few seconds to get that it was normal PQR position posterior to the anus. Maybe add: 1 WT = normal PQR position posterior to the anus or something like that at the bottom of the Table.

Done.

Reviewer 3. This manuscript follows up on a paper published by the Lundquist lab in 2024, which used RNA sequencing of Q-lineage cells in mab-5 gain-and loss-of-function mutants to identify genes under mab-5 transcriptional regulation, in the hope of identifying QL neuroblast lineage-specific genes. In the current manuscript, the authors home in on the atypical kinesin vab-8, frizzled protein lin-17, and ephrin efn-4 as candidate transcriptional targets of mab-5 (originally identified in their 2024 paper). Using genetic analysis, the authors reveal that all three targets function genetically downstream of mab-5. In addition, the authors categorize their contribution to QL descendent cell migration into three distinct phases, with vab-8 having roles in all three phases, lin-17 in phases two and three, while efn-4 is only required for the final cell migration of QL.ap (which will become the PQR neuron) to its terminal location. In support of these data, the authors also performed time-lapse imaging of cell migration defects in vab-8, lin-17, and efn-4 mutants (and others). This revealed some interesting details of QL.ap neural development. If cell migration of QL.ap failed, the cell entered terminal differentiation 1-2 hours early (as defined by the premature extension of a dendrite growth cone), suggesting active suppression of terminal differentiation when the cell body is migrating. Additional cell migration assays and timelapse data on supporting mutants (such as mab-20) nicely round out the study. The authors also publish new mutant alleles that were identified in forward genetics screens to identify novel genes required for QL migration. Overall, this is a thorough and well-executed manuscript with a strong body of genetic analysis, featuring multiple alleles of each gene in question, in all appropriate combinations, and with generous n-numbers, and appropriate statistical analyses. The use of genomic sequencing to clarify the nature of the mab-5(e1751) tandem duplication (an apparent gain-of-function allele) was another nice touch. Note that the RNAseq data upon which this work is based suffers somewhat from overly stringent q-value interpretations/corrections for multiple comparisons, which renders some of the transcript level differences as not-significant when statistically tested as such. Despite this, the authors use of Q-lineage reporter genes in their various genetic backgrounds thoroughly validates the RNAseq data and removes any concern from their RNAseq conclusions.

This is a good point. This is why we “cast a wide net” when testing potential target genes in our genetic assay for function downstream of *mab-5.*

No major revisions are necessary from this reviewer’s point of view. If the following minor comments are addressed, this manuscript will be of a quality and significance appropriate for publication in PLoS Genetics.

Minor comments/typos:

P6 (Abstract) typo, “Surprisingly, failure of stages two and three….”

Done.

P8, first paragraph, “Hox “realizator” genes…” Why not just state “Hox transcriptional targets”?

This is a term commonly used in the literature for Hox gene targets, and we think it puts our work in the broader context of the Hox field.

P9, penultimate paragraph, typo “which acts with efn-4 in prevention…” Also, “posterior QL lineage…)

Done.

P10 last paragraph, “expression of LIN-17/Fz…”

Done.

P12, paragraph 2. Consider changing this sentence to “AQR migration was unaffected in these mutants (Table 2).” to improve readability.

Done.

P13, subheading: state “vab-8, lin-17, and efn-4 lof mutants suppress mab-5 gof”. This will clarify the meaning of this subheading.

Done.

P17, penultimate paragraph, first sentence, “QL.a is divided”; delete “is”.

Done.

P20, penultimate paragraph, last sentence, “are required for to complete…” delete “for”.

Done.

P21, first full paragraph, first sentence, typo: “lin-7” should be “lin-17”. Second sentence, typo: “lin-17(e145)” should be “lin-17(e1456)”.

Done.

P21, last paragraph, typo: “ev754” should be “ev574”. Also, the Culotti et al. 2000 reference should be in parentheses.

Done.

P23, Materials and Methods, Genetics: The authors state that various transgenes generated in their lab were integrated into the genome. Please clarify how this was done, and whether the integrated transgenes were outcrossed prior to use.

Added:

“Transgenes were integrated into the genome using exposure of L4 animals to short-wave ultraviolet radiation for 10 seconds, and subsequent screening of F2 and F3 generations for 100% transmission of the transgene (Mariol *et al.* 2013). Transgenes were outcrossed at least once to N2 before analysis.”

P31, second paragraph: “QL,ap” should be “QL.ap”

Done.

P31 third paragraph, second sentence: “VAB-8 is a conserved…” Consider breaking this sentence into two for improved readability.

Modified as follows:

“VAB-8 is a conserved, atypical Kinesin involved in vesicle trafficking and cell surface localization of the SAX-3 receptor (Watari-Goshima *et al.* 2007; Balseiro-Gomez *et al.* 2022). It is possible that VAB-8 mediates translocation of distinct secreted or transmembrane molecules to the plasma membrane that mediate the distinct steps of QL.a and QL.ap migration.”.

P31, last sentence. Add a period, not a comma.

Done.

P33, figure 1 legend. “lqIs244[Pgcy-32::gfp] -32::GFP” - delete underlined section. Also, please clarify if this is a CFP or GFP reporter gene, and be consistent throughout the text.

Done.

P33 figure 2 legend. **A]** should be A). lqIs220 (last sentence) should be italicized.

Done.

P35 figure 4 legend. “imagijg” should be “imaging”. Figure 5 legend, “hous” should be “hours”

Done.

P37 Acknowledgements: “component” should read “a component”.

Done.

P39, references. The Knop et al., Kratsios et al., Lu et al., Paolillo et al., So et al., references appear incomplete.

Changing the Endnote bibliography style to PLoS seemed to correct this.

P42, table 1. The mab-20 q-value (last row of the table) is less than 0.05 but is reported as not significant. Typo?

It is significant. Corrected.

P43, table 2, suppression experiments: the PQR migration data for mab-5(e1751) doesn’t add up. Typo?

Corrected. 1 position 4, 99 position 5.

P46, table 4. Would it be possible to clarify what “non-position 4” means (RH column)? Is it position 5, or is it anywhere, else other than position 4? Also, last row of table 4, “mom2…” should read “mom-2…”

Added a footnote:

”non-position 4” includes cells that migrated to the wild-type position 5 as well as cells that migrated anteriorly in positions 3, 2, and 1.

Corrected.

Balseiro-Gomez, S., J. Park, Y. Yue, C. Ding, L. Shao *et al.*, 2022 Neurexin and frizzled intercept axonal transport at microtubule minus ends to control synapse formation. Dev Cell 57**:** 1802-1816 e1804.

Gerstein, M. B., Z. J. Lu, E. L. Van Nostrand, C. Cheng, B. I. Arshinoff *et al.*, 2010 Integrative analysis of the Caenorhabditis elegans genome by the modENCODE project. Science 330**:** 1775-1787.

Mariol, M. C., L. Walter, S. Bellemin and K. Gieseler, 2013 A rapid protocol for integrating extrachromosomal arrays with high transmission rate into the C. elegans genome. J Vis Exp**:** e50773.

Meng, L., C. H. Chen and D. Yan, 2016 Regulation of Gap Junction Dynamics by UNC-44/ankyrin and UNC-33/CRMP through VAB-8 in C. elegans Neurons. PLoS Genet 12**:** e1005948.

Sternberg, P. W., K. Van Auken, Q. Wang, A. Wright, K. Yook *et al.*, 2024 WormBase 2024: status and transitioning to Alliance infrastructure. Genetics 227.

Watari-Goshima, N., K. Ogura, F. W. Wolf, Y. Goshima and G. Garriga, 2007 C. elegans VAB-8 and UNC-73 regulate the SAX-3 receptor to direct cell and growth-cone migrations. Nat Neurosci 10**:** 169-176.

Wightman, B., S. G. Clark, A. M. Taskar, W. C. Forrester, A. V. Maricq *et al.*, 1996 The C. elegans gene vab-8 guides posteriorly directed axon outgrowth and cell migration. Development 122**:** 671-682.
